# Supplementary material for: Contributions of distemper control and habitat expansion to the Amur leopard viability
Source: Commun Biol. 2022 Oct 30;5:1153. doi: 10.1038/s42003-022-04127-9 (PMC9618572; doi:10.1038/s42003-022-04127-9)
Supplement: Supplementary file 2 — Supplementary Information [file 42003_2022_4127_MOESM2_ESM.pdf]

## Supplementary information

# Contributions of distemper control and habitat expansion to the Amur leopard viability

**Dawei Wang[1] | Francesco Accatino[2] | James L.D. Smith[3] | Tianming Wang[1]**

[1] Ministry of Education Key Laboratory for Biodiversity Science and Engineering, NFGA Key Laboratory for Conservation Ecology of Northeast Tiger and Leopard & College of Life Sciences, Beijing Normal University, Beijing, 100875, China

[2] UMR SADAPT, INRAE, AgroParisTech, Université Paris-Saclay, 22 place de l'agronomie, CS 80022, 91120 PALAISEAU Cedex, France

[3] Department of Fisheries, Wildlife and Conservation Biology, University of Minnesota, St. Paul, MN 55108, USA

Correspondence

Tianming Wang

Email: wangtianming@bnu.edu.cn

## *Supplementary Method 1: Detailed canine distemper virus (CDV) transmission supplementary information for Outbreak*

**1. Software information**

**2. Baseline Disease Parameters for CDV Epidemiological Mode**

*i.* Pre-susceptible
*ii.* Susceptible
*iii.* Exposed and infected
*iv.* Infectious
*v.* Recovered and resistant

## *Supplementary Method 2: Supplementary information of Amur leopard population dynamics for Vortex*

**1. Software information**

**2. Baseline Parameters for Population Dynamics Mode**

*i.* Scenario setting
*ii.* Species description
*iii.* Reproductive system
*iv.* Reproductive rate
*v.* Mortality rates
*vi.* Catastrophes
*vii.* Population size

## *Supplementary Method 3: Component model connector-MetaModel Manager*

**1. Software information**

**2. Settings for MetaModel Manager**

## *References*

## *Supplementary Tables*

*i.* Table S6
*ii.* Table S7
*iii.* Table S8

***Supplementary Method 1: Detailed canine distemper virus (CDV) transmission supplementary information for Outbreak***

## 1. Software information

Outbreak was developed to better guide disease management strategies for endangered species by assessing the impacts of infectious disease on population viability in combination with particular demographic parameters in a probabilistic framework.

Given the new deadly threat in the Amur leopard population, CDV, we developed an individual-based epidemiological model based on a complete evaluation of available field and laboratory data that simulated CDV spread through the Amur leopard population on the Sino-Russian border. Outbreak version 2.11.0[1] software was used to simulate basic epidemiological processes and report on survival status at a daily time step. For further information on how to use the software, see the Outbreak User's Manual Version 1.1[2], which is available at https://scti.tools/outbreak/. Based on the population information provided by the Amur leopard population model, the epidemiological model calculates the following: (i) the number of individuals who become carriers; (ii) the number of individuals infected and spreading the virus; and (iii) the number of individuals that die due to CDV. The Outbreak model is described in detail below.

## 2. Baseline Disease Parameters for CDV Epidemiological Mode

### *i.* Pre-susceptible

All individuals from birth to the earliest age of susceptibility are considered in the pre-susceptible state (P).

(1) Proportion that never becomes susceptible = 0

(2) Earliest age of susceptibility (days) = IUNFORM (1;2)

IUNIFORM (A; B) means a random integer from A to B (the same below).

There is no evidence that newborn Amur leopard cubs are not susceptible to CDV. Therefore, cubs are susceptible after birth and may become infected within the first couple days.

(3) Transmission probability from an infectious (I) mother to a newborn (Probability 0—1) = 1

Some previous studies have shown that CDV has not been detected in the dead cubs of female large cats who have been infected with CDV and died[3]. However, decay may affect the sensitivity of detection to a certain extent, and females infected with CDV are highly likely to transmit the virus to their cubs. Cubs that lose their mother have difficulty surviving, so we set the transmission probability to 1.

(4) Do resistant (R) females pass immunity to their offspring at birth? Yes

(5) Days for maternally derived immunity to protect an offspring = IUNIFORM (150;180)

Recovered leopards will have immunity for the rest of their lives. The duration of lactation of leopard cubs is approximately five to six months, during which time the cubs can obtain antibodies from their mother's milk.

### *ii.* Susceptible

A susceptible (S) individual can become exposed and infected (E) given exposure to a disease agent. The transition from S to E can occur by encountering an infectious (I) individual or coming into touch with an environmental disease source (including predation).

(1) Average encounter rate per day with an outside disease source = 1

(2) When an environmental source is encountered, the probability of transmission = 0.00078904 or 0.000660291 (absence of dog control and dog control, respectively)

Contact with dogs and wild carnivores is classified as contact with pathogenic environments. Because there are two types of contact for the leopard, the contact probability and prey's prevalence of the two contact types are different. Therefore, we integrated the parameters. We set the average encoder rate per day to 1. According to the average number of small and medium-sized prey captured by Amur leopards calculated following Sugimoto et al. (2016)[4] and the probability of CDV infection of each species in Table S1, the average annual probability of CDV infection of Amur leopards in the susceptible state during an epizootic cycle (5 years) was calculated to be 0.288 and 0.241, respectively. The probability of infection by pathogenic environment in the absence of dog control = 0.288/365 = 0.00078904. The probability of infection by pathogenic environment in the case of dog control = 0.241/365 = 0.000660291.

**Table S1** The small- and medium-sized carnivorous prey composition of Amur leopards and CDV infection probability of prey. W = prey weight (kg); R = relative biomass consumed; N = mean number of preys eaten per leopard per year (rounded).

| Carnivorous prey species | W | R | N | Probability of CDV infection | | |
|---|---|---|---|---|---|---|
| | | | | Low risk | High risk | Source |
| Badger (*Meles meles*) | 6 | 2.7% | 9 | 0.6% | 5.0% | Pavlacik et al. (2007)[5] |
| Red fox (*Vulpes vulpes*) | 5 | 0.3% | 1 | 2.0% | 4.0% | Nouvellet et al. (2013)[6] |
| Leopard cat (*Felis bengalensis*) | 4 | 1.6% | 8 | 0.8% | 3.6% | Ikeda et al. (2001)[7]; Sacristán et al. (2019)[8] |
| Domestic dog (*Canis familiaris*) | 30 | 4.5% | 3 | 0.77% | 5.0% | Gilbert et al. (2020)[9] Rautenbach et al. (1991)[10] |
| Others | - | 3.7% | - | 0 | 0 | - |

(3) Number of individuals who an individual in state I encounters per day = 0.05

Amur leopards generally act alone, and they only have contact with other individuals when they are in mating. There are 2-3 interactions per month between individuals during the mating period. However, when the female leopard is pregnant or with her cub, she will try to avoid contact with the male leopard. Therefore, on average, we conservatively set the effective interaction number to 1-2 per month. Therefore, the number of individuals encountered every day was approximately 0.05.

(4) When an I individual encounters an S individual, the probability of transmission = 0.288 or 0.241 (absence of dog control and dog control, respectively)

The probability of CDV transmission among leopards was estimated as the probability of leopards contracting CDV from the environment.

### *iii.* Exposed and infected

An exposed (E) individual is one who has encountered an infected individual or an outside disease source and contracted the disease agent but has not yet become infectious. Such individuals can remain in this state for an extended period that is determined by the incubation, or latent period, of the disease.

Duration of the incubation period (latency) in days = IUNIFORM (2;7)

### *iv.* Infectious

An infectious individual is one that is actively shedding the disease agent and is, therefore, capable of transmitting the disease to another individual.

In this model, an infectious individual may:

- recover without immunity (move to S);

- recover with immunity (move to R);
- die.

(1) Proportion of I individuals that remain infectious indefinitely = 0

(2) Duration of the infectious period in days = IUNIFORM (30;60)

The duration of CDV infection is highly variable and depends on the susceptibility of the host species, immune capacity, and virus strain[11]. Most cases last one to two months[12].

(3) Probability of recovering and becoming resistant = 0.6

(4) Probability of returning to the susceptible state = 0

(5) Probability of dying from the infection = 0.4

There is no precise measurement of CDV-induced leopard mortality. The fatality rate after infection with CDV is estimated to be 40% based on published case reports of large cats[12].

## *v.* Recovery and resistance

If a leopard has recovered from a CDV infection, it will not be infected again.

R1 – Proportion of R individuals acquiring permanent immunity = 1

***Supplementary Method 2: Supplementary information of Amur leopard population dynamics for Vortex***

## 1. Software information

Vortex is an individual-based model that keeps track of each animal's sex, age, and parentage. Demographic events (birth, sex determination, mating, dispersion, and death) are represented by assessing whether any of the events occur for each animal in each year of the simulation[13], which is used to simulate population changes over a given period, allowing the probability of extinction or population decline to be assessed based on available data and models. We used software Vortex, version 10.5.5[14] to build a population model to complete the viability analysis of the Amur leopard population. Referring to the Vortex 10 User's Manual[15] for the use of the software, the above content is available at https://scti.tools/vortex/. The baseline model parameters required by Vortex are as follows.

## 2. Baseline Parameters for Population Dynamics Mode

### *i.* Scenario setting

The basic setup of the simulation program.

(1) Extinction definition: Only 1 sex remains

(2) Number of populations: 1

### *ii.* Species description

Describe a few questions about population dynamics.

(1) Lethal equivalents = 3.14

The number of lethal equivalents can be interpreted as the number of deaths that would be expected in a group of hypothetical individuals if each individual carried one

deleterious allele in a homozygous state (i.e., the group contains as many individuals as there are deleterious alleles[16]). Hence, one lethal equivalent can correspond to a lethal allele at one locus or to several mildly deleterious alleles at several loci. In there the number of lethal equivalents is used to describe the severity of inbreeding decline in a simulated population. According to the study of 40 captive mammal populations by Ralls et al. (1988)[17], the median lethal equivalent was 3.14. Although some scholars believe that the impact of inbreeding on most wild animal populations is underestimated by 3.14, there is no study that provides the exact lethal equivalent value of wild Amur leopards, so we conservatively set the lethal equivalent value to 3.14 in the baseline model.

(2) Percent due to recessive lethal alleles = 50%

A plausible value that is consistent with data on Drosophila and a few other species that have been well studied well is 50%.

### *iii.* Reproductive system

This part of the parameter setting was mainly based on the comprehensive consideration of the existing studies on the Amur leopard.

(1) Breeding system: Polygamous

(2) Age of first offspring (female/male) = 3/3

(3) Maximum age of reproduction = 12

(4) Maximum lifespan = 12

(5) Maximum number of broods per year = 1

(6) Maximum number of progenies per brood = 3

(7) Sex ratio at birth (expressed as % of males) = 50

(8) Length of time cubs depend on their mother (years) = 1

The duration of pregnancy of Amur leopard is 90-95 days. The cubs will not leave the female leopard before 12 months after birth. During this period, the cubs cannot survive independently. Therefore, we set the time that the cubs depend on their mother as 1 year.

### *iv.* Reproductive rate

This part of the parameter setting was mainly based on the comprehensive consideration of the existing studies on the Amur leopard.

(1) % of Males in breeding pool = 70

(2) % of adult females successfully breeding = (IS1 = 0) *70

For % of adult females successfully breeding, we specify the mean percentage of adult females that breed in a given year (or, stated another way, the probability that a given adult female will successfully produce offspring in a given year). "IS1 = 0" denotes the females without cubs to raise. The parameter settings of (1) and (2) were based on the comprehensive consideration of Wilkinson and O'Regan (2003)[18].

(3) Litter size: See Table S2

**Table S2** Litter size distribution data used in the baseline model, based on Wilkinson and O'Regan (2003)[18].

| Litter size | Proportion (%) |
|---|---|
| 1 offspring | 30 |
| 2 offspring | 60 |
| 3 offspring | 10 |

## *v.* Mortality rates

Due to the lack of mortality data of the Amur leopard, we referred to the mortality data of the African leopard (Panthera pardus pardus) from 2002 to 2007 in the Mkhuze Game Reserve, South Africa, where the anthropogenic disturbance is similar to that in the Sino-Russian border area. Balme et al. (2010) captured 17 leopards in Mkhuze between 2002 and 2007[19], equipped them with a VHF radio-collar or a GPS collar with a UHF uplink and calculated the mortality rate of African leopards at various ages (Table S3) and the proportion of deaths attributed to different causes (Table S4). Because no fatalities of adult females were detected throughout the monitoring period in the Mkhuze Game Reserve and no monitoring of 0- to 1-year-old cubs was carried out, we assessed the mortality rate of adult females to be 5% and the mortality rate of 0- to 1-year-old cubs to be 30% after consulting with experts in the baseline scenario (Table S5).

**Table S3** Annual leopard mortality rates, Mkhuze Game Reserve, South Africa, 2002 to 2007

| Age/sex class | Mortality rates |
|---|---|
| Cub (0-1) | - |
| Subadult male (1-3) | 0.195 |
| Subadult female (1-3) | 0.118 |
| Adult Male (>3) | 0.082 |
| Adult Female (>3) | - |

**Table S4** Cause-specific annual mortality rates and number of deaths for leopards in the Mkhuze Game Reserve, South Africa, 2002 to 2007

| Cause of mortality | Mortality rates | Deaths |
|---|---|---|
| Nature | 0.042 | 1 |
| Anthropogenic | 0.042 | 1 |
| Unknown | 0.042 | 1 |

**Table S5** The mortality rate of Amur leopards under the baseline scenario

| Age/sex class | Mortality rates |
|---|---|
| Cub (0-1) | 0.3000 |
| Subadult male (1-3) | 0.1950 |
| Subadult female (1-3) | 0.1180 |
| Adult male (>3) | 0.0820 |
| Adult female (>3) | 0.0500 |

## *vi.* **Population size**

(1) Initial population size = 87

(2) Carrying capacity (K) = 56 (adult females)

*Supplementary Method 3: Component model connector-MetaModel Manager*

## 1. Software information

Metamodel Manager is a highly adaptable program that connects existing models (or new ones built to fill specific gaps) via the various interacting components of a complex system by controlling the order in which the component models are called and the flow of information between them. Each model can be used to compute or simulate changes to its variables and processes while being informed about changes to other parts of the system using shared descriptors of the system at the three levels: individual, population, and system (or 'global') level variables.

We used the software Metamodel Manager, version 1.0.6[20] to connect and set the time interval of the CDV epidemiological model and the Amur leopard population demographic model. Referring to Metamodel Manager User's Manual Version 1.1[21] for the use of the software, the above content is available at https://scti.tools/metamodelmanager/.

## 2. Settings for MetaModel Manager

(1) Time interval for the CDV epidemiological model = 1 day

(2) Time interval for the Amur leopard population dynamics model = 1 year

(3) MetaModel simulation cycles = 50

(4) Number of iterations = 1000

## References


1. Lacy, R. C., Pollak, J. P., Miller, P. S., Hungerford, L. & Bright, P. *Outbreak. Version 2.10*. (2020).
2. Pacioni, C., Sullivan, S., Lees, C. M., Miller, P. S. & Lacy, R. C. Outbreak user's manual. Version 1.1. (2020).
3. Gilbert, M. *et al.* Canine distemper virus as a threat to wild tigers in Russia and across their range. *Integr. Zool.* 10, 329–343 (2015).
4. Sugimoto, T., Aramilev, V. V., Nagata, J. & McCullough, D. R. Winter food habits of sympatric carnivores, Amur tigers and Far Eastern leopards, in the Russian Far East. *Mamm. Biol.* 81, 214–218 (2016).
5. Pavlacik, L., Celer, V., Koubek, P. & Literak, I. Prevalence of canine distemper virus in wild mustelids in the Czech Republic and a case of canine distemper in young stone martens. *Vet. Med. (Praha)* (2007).
6. Nouvellet, P. *et al.* Rabies and canine distemper virus epidemics in the red fox population of northern Italy (2006-2010). *PLOS ONE* 8, e61588 (2013).
7. Ikeda, Y. *et al.* Seroprevalence of canine distemper virus in cats. *Clin. Diagn. Lab. Immunol.* 8, 641–644 (2001).
8. Sacristán, I. *et al.* Molecular and serological survey of carnivore pathogens in free-roaming domestic cats of rural communities in southern Chile. *J. Vet. Med. Sci.* 81, 1740–1748 (2019).
9. Gilbert, M. *et al.* Distemper, extinction, and vaccination of the Amur tiger. *Proc. Natl. Acad. Sci.* 117, 31954–31962 (2020).
10. Rautenbach, G. H., Boomker, J. & de Villiers, I. L. A descriptive study of the canine population in a rural town in southern Africa. *J. S. Afr. Vet. Assoc.* 62, 158–162 (1991).
11. Greene, C. Infectious diseases of the dog and cat. *Aust. Vet. J.* 77, 194–194 (2008).
12. Gilbert, M. *et al.* Estimating the potential impact of canine distemper virus on the Amur tiger population (*Panthera tigris altaica*) in Russia. *PLOS ONE* 9, e110811 (2014).
13. Bradshaw, C. J. A. *et al.* Novel coupling of individual-based epidemiological and demographic models predicts realistic dynamics of tuberculosis in alien buffalo. *J. Appl. Ecol.* 49, 268–277 (2012).
14. Lacy, R. C. & Pollak, J. P. *Vortex: A stochastic simulation of the extinction process. Version 10.4*. (2021).
15. Lacy, R. C., Miller, P. S. & Traylor-Holzer, K. Vortex 10 User's Manual. (2021).
16. Morton, N. E., Crow, J. F. & Muller, H. J. An estimate of the mutational damage in man from data on consanguineous marriages. *Proc. Natl. Acad. Sci.* 42, 855–863 (1956).
17. Ralls, K., Ballou, J. D. & Templeton, A. Estimates of lethal equivalents and the cost of inbreeding in mammals. *Conserv. Biol.* 2, 185–193 (1988).
18. Wilkinson, D. M. & O'Regan, H. J. Modelling differential extinctions to understand big cat distribution on Indonesian islands. *Glob. Ecol. Biogeogr.* 12, 519–524 (2003).
19. Balme, G. A., Slotow, R. & Hunter, L. T. B. Edge effects and the impact of non-protected areas in carnivore conservation: leopards in the Phinda–Mkhuze Complex, South Africa. *Anim. Conserv.* 13, 315–323 (2010).
20. Pollak, J. P. & Lacy, R. C. *Metamodel manager. Version 1.0.6*. (2020).
21. Raboy, B.E., Lacy, R.C., Callicrate, T., Lees, C.M. Metamodel manager. user's manual. Version 1.1. *SCTI* https://scti.tools/downloads/#SoftwareAndManuals (2018).

**Table S6** Details of the indices of the Amur leopard population under different management measures for three values of inbreeding depression. R (SD): mean (standard deviation) of population exponential growth rate averaged across all years and iterations; N (SD): mean (standard deviation) number of leopards at year 50; GD (SD): initial gene diversity (heterozygosity) remaining in extant populations at year 50; PE: the probability of extinction, defined as only 1 sex remaining at year 50; TE: mean time of extinction (in years) in those iterations that did have an extinction.

| LEs | Scenario | R (SD) | N (SD) | GD (SD) | PE | TE |
|---|---|---|---|---|---|---|
| 3.14 | Baseline | -0.011 (0.101) | 46 (41) | 0.763 (0.131) | 0.202 | 78 |
| | Control dogs | -0.003 (0.088) | 73 (47) | 0.797 (0.121) | 0.092 | 77 |
| | Vaccination | 0.001 (0.078) | 81 (41) | 0.819 (0.079) | 0.032 | 78 |
| | Expand habitat | -0.011 (0.102) | 57 (53) | 0.776 (0.135) | 0.193 | 77 |
| 6.29 | Baseline | -0.030 (0.125) | 9 (16) | 0.702 (0.162) | 0.618 | 78 |
| | Control dogs | -0.022 (0.11) | 20 (27) | 0.752 (0.137) | 0.421 | 80 |
| | Vaccination | -0.017 (0.103) | 26 (28) | 0.747 (0.139) | 0.249 | 83 |
| | Expand habitat | -0.031 (0.124) | 10 (20) | 0.710 (0.154) | 0.616 | 75 |
| 12.26 | Baseline | -0.058 (0.157) | 0 (0) | 0(0) | 0.999 | 56 |
| | Control dogs | -0.053 (0.153) | 0 (0) | 0(0) | 0.999 | 61 |
| | Vaccination | -0.050 (0.151) | 0 (0) | 0(0) | 0.999 | 65 |
| | Expand habitat | -0.058 (0.157) | 0 (0) | 0(0) | 0.999 | 56 |

**Table S7** Details of indices of the Amur leopard population under different management measures for three values of inbreeding depression. R (SD): mean (standard deviation) of population exponential growth rate averaged across all years and iterations; N (SD): mean (standard deviation) number of leopards at year 50; GD (SD): initial gene diversity (heterozygosity) remaining in extant populations at year 50; PE: the probability of extinction, defined as only 1 sex remaining at year 50; TE: mean time of extinction (in years) in those iterations that did have an extinction; Scenario A: controlling the domestic dogs around the habitat; Scenario B: low-coverage vaccinating for leopards; Scenario C: expanding habitat for leopards.

| LEs | Scenario | R (SD) | N (SD) | GD (SD) | PE | TE |
|---|---|---|---|---|---|---|
| 3.14 | Baseline | -0.011 (0.101) | 46 (41) | 0.763 (0.131) | 0.202 | 78 |
| | A + C | -0.002 (0.085) | 96 (64) | 0.822 (0.11) | 0.101 | 77 |
| | B + C | 0 (0.078) | 100 (57) | 0.821 (0.108) | 0.03 | 76 |
| | A + B | 0.007 (0.07) | 107 (37) | 0.845 (0.078) | 0.009 | 71 |
| | A + B + C | 0.008 (0.068) | 139 (53) | 0.856 (0.087) | 0.009 | 68 |
| 6.29 | Baseline | -0.030 (0.125) | 9 (16) | 0.702 (0.162) | 0.618 | 78 |
| | A + C | -0.021 (0.108) | 26 (35) | 0.762 (0.135) | 0.385 | 79 |
| | B + C | -0.015 (0.099) | 35 (36) | 0.764 (0.147) | 0.217 | 84 |
| | A + B | -0.008 (0.087) | 46 (34) | 0.794 (0.114) | 0.106 | 87 |
| | A + B + C | -0.006 (0.083) | 64 (47) | 0.816 (0.116) | 0.097 | 85 |
| 12.26 | Baseline | -0.058 (0.157) | 0 (0) | 0 (0) | 0.999 | 56 |
| | A + C | -0.054 (0.152) | 0 (0) | 0 (0) | 0.999 | 61 |
| | B + C | -0.050 (0.149) | 0 (0) | 0.356 (0.375) | 0.998 | 65 |
| | A + B | -0.046 (0.146) | 0 (0) | 0.431 (0.503) | 0.997 | 70 |
| | A + B + C | -0.046 (0.146) | 0 (1) | 0.501 (0.313) | 0.992 | 71 |

**Table S8** Details of indices of the Amur leopard population under different low-coverage vaccinations (different numbers of vaccinated individuals per year) for three values of inbreeding depression. R (SD): mean (standard deviation) of population exponential growth rate averaged across all years and iterations; N (SD): mean (standard deviation) number of leopards at year 50; GD (SD): initial gene diversity (heterozygosity) remaining in extant populations at year 50; PE: the probability of extinction, defined as only 1 sex remaining at year 50; TE: mean time of extinction (in years) in those iterations that did have an extinction; Scenario A: controlling the domestic dogs around the habitat; Scenario B: low-coverage vaccinating for leopards; Scenario C: expanding habitat for leopards.

| LEs | Scenario | R (SD) | N (SD) | GD (SD) | PE | TE |
|---|---|---|---|---|---|---|
| 3.14 | 0 | -0.011 (0.101) | 46 (41) | 0.763 (0.131) | 0.202 | 78 |
| | 2 | -0.006 (0.093) | 61 (44) | 0.784 (0.131) | 0.122 | 76 |
| | 4 | -0.002 (0.085) | 74 (44) | 0.796 (0.116) | 0.06 | 77 |
| | 6 | 0.001 (0.078) | 81 (41) | 0.819 (0.079) | 0.032 | 78 |
| | 8 | 0.003 (0.076) | 90 (39) | 0.829 (0.081) | 0.018 | 78 |
| | 10 | 0.005 (0.073) | 99 (39) | 0.838 (0.08) | 0.016 | 82 |
| 6.29 | 0 | -0.030 (0.125) | 9 (16) | 0.702 (0.162) | 0.618 | 78 |
| | 2 | -0.024 (0.116) | 16 (22) | 0.713 (0.156) | 0.428 | 81 |
| | 4 | -0.020 (0.108) | 21 (25) | 0.738 (0.144) | 0.344 | 82 |
| | 6 | -0.017 (0.103) | 26 (28) | 0.747 (0.139) | 0.249 | 83 |
| | 8 | -0.014 (0.097) | 33 (31) | 0.769 (0.116) | 0.2080 | 84 |
| | 10 | -0.012 (0.093) | 37 (31) | 0.773 (0.125) | 0.124 | 84 |
| 12.26 | 0 | -0.058 (0.157) | 0 (0) | 0 (0) | 0.999 | 56 |
| | 2 | -0.054 (0.155) | 0 (0) | 0 (0) | 0.999 | 60 |
| | 4 | -0.052 (0.153) | 0 (0) | 0 (0) | 0.999 | 62 |
| | 6 | -0.050 (0.151) | 0 (0) | 0 (0) | 0.999 | 65 |
| | 8 | -0.048 (0.148) | 0 (0) | 0 (0) | 0.999 | 67 |
| | 10 | -0.047 (0.148) | 0 (0) | 0.598 (0.038) | 0.998 | 69 |
