# Supplementary material for: Contributions of distemper control and habitat expansion to the Amur leopard viability
Source: Commun Biol. 2022 Oct 30;5:1153. doi: 10.1038/s42003-022-04127-9 (PMC9618572; doi:10.1038/s42003-022-04127-9)
Supplement: Supplementary file 3 — Reporting Summary [file 42003_2022_4127_MOESM3_ESM.pdf]

nature portfolio

Corresponding author(s): Tianming Wang

Last updated by author(s): Sep 21, 2022

Reporting Summary

Nature Portfolio wishes to improve the reproducibility of the work that we publish. This form provides structure for consistency and transparency in reporting. For further information on Nature Portfolio policies, see our Editorial Policies and the Editorial Policy Checklist.

Statistics

For all statistical analyses, confirm that the following items are present in the figure legend, table legend, main text, or Methods section.

n/a	Confirmed	
☒	☐	The exact sample size (*n*) for each experimental group/condition, given as a discrete number and unit of measurement
☒	☐	A statement on whether measurements were taken from distinct samples or whether the same sample was measured repeatedly
☒	☐	The statistical test(s) used AND whether they are one- or two-sided<br>*Only common tests should be described solely by name; describe more complex techniques in the Methods section.*
☒	☐	A description of all covariates tested
☒	☐	A description of any assumptions or corrections, such as tests of normality and adjustment for multiple comparisons
☒	☐	A full description of the statistical parameters including central tendency (e.g. means) or other basic estimates (e.g. regression coefficient) AND variation (e.g. standard deviation) or associated estimates of uncertainty (e.g. confidence intervals)
☒	☐	For null hypothesis testing, the test statistic (e.g. *F*, *t*, *r*) with confidence intervals, effect sizes, degrees of freedom and *P* value noted<br>*Give P values as exact values whenever suitable.*
☒	☐	For Bayesian analysis, information on the choice of priors and Markov chain Monte Carlo settings
☒	☐	For hierarchical and complex designs, identification of the appropriate level for tests and full reporting of outcomes
☒	☐	Estimates of effect sizes (e.g. Cohen's *d*, Pearson's *r*), indicating how they were calculated

Our web collection on statistics for biologists contains articles on many of the points above.

Software and code

Policy information about availability of computer code

Data collection	The parameters used in this study were sourced from published literature, but no software was used for data collection.
Data analysis	The simulations in this study used the software Vortex, Outbreak, MetaModel Manager, available at https://scti.tools/downloads/ #SoftwareAndManuals

For manuscripts utilizing custom algorithms or software that are central to the research but not yet described in published literature, software must be made available to editors and reviewers. We strongly encourage code deposition in a community repository (e.g. GitHub). See the Nature Portfolio guidelines for submitting code & software for further information.

Data

Policy information about availability of data

All manuscripts must include a data availability statement. This statement should provide the following information, where applicable:

- Accession codes, unique identifiers, or web links for publicly available datasets
- A description of any restrictions on data availability
- For clinical datasets or third party data, please ensure that the statement adheres to our policy

All data generated or analysed during this study are included in this published article (and its supplementary information files).

Field-specific reporting

Please select the one below that is the best fit for your research. If you are not sure, read the appropriate sections before making your selection.

☐ Life sciences ☐ Behavioural & social sciences ☒ Ecological, evolutionary & environmental sciences

For a reference copy of the document with all sections, see nature.com/documents/nr-reporting-summary-flat.pdf

Ecological, evolutionary & environmental sciences study design

All studies must disclose on these points even when the disclosure is negative.

Study description	For the first time, we use a population viability analysis (PVA) metamodel to provide a comprehensive assessment of factors threatening the small Amur leopard population along the China-Russia border, in which, demography, genetics, and disease are modelled synergistically.
Research sample	The research sample studied was the Amur leopard population on the China-Russia border, which is the only remaining wild Amur leopard population in the world and is at high risk of extinction. The parameters of the population were sourced from published literature, and the sources are shown in the text.
Sampling strategy	The study is to simulate the future trend of the Amur leopard population and does not involve sampling strategy.
Data collection	Dawei Wang collected the parameters needed for the simulation by reviewing the literature about the Amur leopard and other subspecies of leopard.
Timing and spatial scale	Parameter collection from July 2021 to January 2022
Data exclusions	No data exclusion involved
Reproducibility	All parameters required for the simulations in this study and the download addresses of the simulation softwares used are detailed in the text or in the supplementary information files.
Randomization	The simulation results have a certain randomness, expressed as the mean ± standard deviation.
Blinding	The study is about using the metamodel to simulate the future development trend of the population and does not involve blinding.

Did the study involve field work? ☐ Yes ☒ No

Reporting for specific materials, systems and methods

We require information from authors about some types of materials, experimental systems and methods used in many studies. Here, indicate whether each material, system or method listed is relevant to your study. If you are not sure if a list item applies to your research, read the appropriate section before selecting a response.

Materials & experimental systems

n/a	Involved in the study	
☒	☐	Antibodies
☒	☐	Eukaryotic cell lines
☒	☐	Palaeontology and archaeology
☒	☐	Animals and other organisms
☒	☐	Human research participants
☒	☐	Clinical data
☒	☐	Dual use research of concern

Methods

n/a	Involved in the study	
☒	☐	ChIP-seq
☒	☐	Flow cytometry
☒	☐	MRI-based neuroimaging
